# Digital Holography Using Harmonic Generation from Solids for Reconstruction of Subwavelength Nanostructures

**DOI:** 10.1021/acsphotonics.5c02501

**Published:** 2026-01-31

**Authors:** Leo Guery, Falco Bijloo, Peter M. Kraus

**Affiliations:** † 530573 Advanced Research Center for Nanolithography, Science Park 106, 1098 XG Amsterdam, Netherlands; ‡ Department of Physics of Information in Matter and Center for Nanophotonics, 55952 NWO-I Institute AMOLF, Science Park 104, 1098 XG Amsterdam, Netherlands; § Vrije Universiteit Amsterdam, De Boelelaan 1105, 1081 HV Amsterdam, Netherlands

**Keywords:** digital holographic microscopy, third-harmonic generation, high-harmonic generation, nanoscale metrology, interferometry, critical dimension inspection, ultrafast optics

## Abstract

Digital holographic microscopy (DHM) is a successful technique frequently used to assess the phase in imaging experiments. Combining DHM with nonlinear generation opens the possibility of measuring phases in nonlinear processes such as high-harmonic generation and characterizing nanostructures with an increased sensitivity. In this paper, we demonstrate that the combination of DHM and harmonic generation from solids can be used to reliably perform 3D reconstructions of samples and also investigate structural parameters of subwavelength periodic structures with improved accuracy. We were able to discriminate gratings etched in silicon, with only a few tens of nanometers change in critical dimension, down to a pitch of 400 nm, which is well below the wavelength of the near-infrared (NIR) probing laser source. This technique can in principle be used with all high-harmonic-emitting materials and is expected to reach even larger gains in resolution by probing higher-order harmonics. These results pave the way for sensing of subwavelength structures via nonlinear light generation, for instance, in the semiconductor industry.

## Graphical abstract

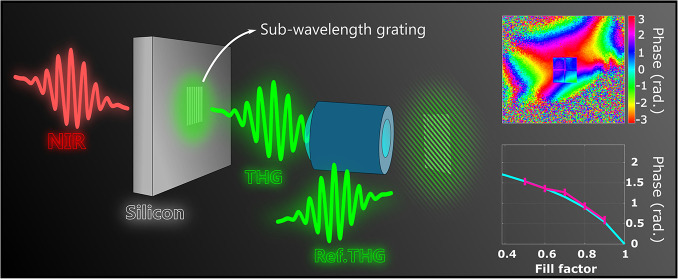

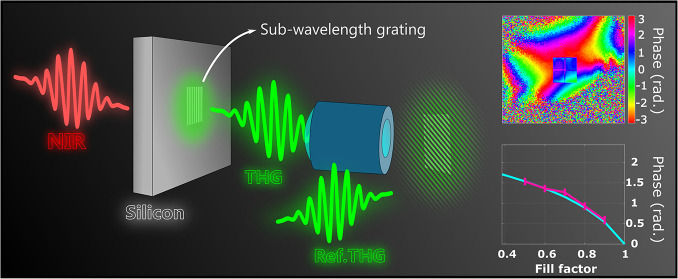

## Introduction

While conventional microscopy only measures intensity patterns, the phase is crucial to conduct an accurate 3D reconstruction. Measuring both amplitude and phase enables reconstruction of both real and imaginary parts of the refractive index, thus enhancing contrast between different materials compared with images obtained by only considering amplitude. More generally, the measuring phase is a necessity in order to obtain a complete picture of light–matter interactions in a sample.

A number of well-established techniques allow for phase sensitive imaging, for instance, holography, in all its variants (including DHM),^1–3^ ptychography,^4^ and coherent-diffraction imaging.^5^ The latter two are usually recorded in Fourier space and thus generally preferred when performing measurements where no optics for reimaging are available, i.e., for wavelengths shorter than the ultraviolet range. For longer wavelengths, real-space digital holography is both a fast and reliable way to recover phase, as it does not rely on phase retrieval algorithms, which can sometimes lead to errors in the reconstruction and involve an extended computation time.

A conventional DHM measurement relies on combining the image of a sample obtained by using a microscope with a reference wave at the same wavelength so that both interfere in the plane of a detector. These interferences encode both the amplitude and phase of the electrical field at the sample plane position, which can be recovered by applying straightforward numerical operations.^6^ The measurement scheme that we present in this paper relies on the same core principle. The main difference lies in the nature of the light emitted by the sample, which is at a harmonic order of the fundamental light illuminating the sample.

Using nonlinear light–matter interactions for developing new imaging techniques has received a lot of attention as the availability of suitable ultrafast laser sources has grown. Both second and third-harmonic generation (SHG and THG) microscopy have found applications in a large variety of research fields, such as biological imaging and^7,8^ 2D surface inspection,^9^ and have been further developed to enable super-resolution microscopy below the diffraction limit.^10^ A central advantage of nonlinear microscopy resides in the possibility to generate light in localized areas of interest in a sample, thus enhancing the contrast of an image and reducing the necessity to use chemical labels.

Nonlinear holography is starting to draw some attention as well. Early work by Ye Pu and Hsieh C. in 2008 describes an experiment in which SHG emitted by a collection of nanoscatterers is used to form a hologram, allowing to trace back the 3D positions of the particles.^11,12^ Similar results were achieved by Schaffer et al.^13^ as well as measurements of biological samples in a second paper.^14^ More recent work by Farah et al.^15^ focused on synthetic spatial aperture THG hologram reconstructions, emphasizing aberration removal using algorithms. Higher-order harmonics were also investigated to perform coherent-diffraction imaging.^16,17^

The principle of holography can also be applied to shape the light wavefront in a desired way by making a light source interact with a well-defined nanostructure. Patterned samples used for that purpose are often referred to as metasurfaces and have a wide variety of applications.^18^ Significant efforts have recently been put in designing resonant metasurfaces allowing to control high-harmonic generation (HHG) emission, wherever it is to increase conversion efficiency,^9^ shape the emitted wavefront,^19^ or control polarization of the harmonic light.^20^

With this paper, we pioneer nonlinear DHM on solid nanostructures and demonstrate two applications: first, we show how holograms can be measured through transmission of thick and opaque materials with an enhanced resolution; second, we show how nonlinear DHM can be used to characterize periodic subwavelength structures.

## Methods

### Analytical Framework for Transmission THG

In perturbative nonlinear optics, the polarization at the third-harmonic frequency 3ω induced by an external field oscillating at ω is given by^21^

1 $${\mathrm{P̃}}_{3\omega }\left(z,t\right)=\epsilon_{0}\chi^{\left(3\right)}{Ẽ}_{\omega }^{3}\left(z,t\right)$$

where ϵ_0_ is the free space permittivity and χ^(3)^ is the third-order nonlinear susceptibility of the material. For the excitation field, we consider a plane wave propagating along the *z* axis. Leaving the temporal dependence aside, we can write

2 $$E_{\omega }\left(z\right)=E_{0}\left(z\right)e^{i\left(k_{\omega }z+\phi_{\omega }\right)}$$

with *E*_0_ being the absolute value of the field and *k*_ω_ its wave vector. Substituting this expression in eq 1, we get

3 $$P_{3\omega }\left(z\right)=\epsilon_{0}\chi^{\left(3\right)}E_{0}e^{i\left(3k_{\omega }z+3\phi_{\omega }\right)}$$

We are interested in the third harmonic emitted through the transmission of a silicon sample of width *L*. At the backside exit of the material, it can be expressed as the sum of contributions emitted from each slab along *z*

4 $$E_{3\omega }\left(z\right)\propto \int_{0}^{L}P_{3\omega }\left(z\right)e^{ik_{3\omega }\left(L-z\right)}e^{-\alpha \left(L-z\right)}dz$$

where the first exponential term accounts for propagation of the third harmonic after generation and the second one for losses in the material, with α being the absorption coefficient for the third harmonic. Substituting *P*_3ω_(*z*) by its expression in eq 3, we get

5 $$\begin{array}{l} E_{3\omega }\left(z=L\right)=\int_{0}^{L}\epsilon_{0}\chi^{\left(3\right)}E_{0}^{3}e^{i3\left(k_{\omega }z+\phi_{\omega }\right)}e^{ik_{3\omega }\left(L-z\right)}e^{-\alpha \left(L-z\right)}dz\\ =\epsilon_{0}\chi^{\left(3\right)}E_{0}^{3}e^{i\left(k_{3\omega }L+3\phi_{\omega }\right)}\int_{0}^{L}e^{i\Delta \mathrm{kz}}e^{-\alpha \left(L-z\right)}dz \end{array}$$

where we recognize the phase matching term Δ*k*

6 $$\Delta k=3k_{\omega }-k_{3\omega }$$

Solving the integral, we obtain

7 $$E_{3\omega }\left(z=L\right)=\epsilon_{0}\chi^{\left(3\right)}E_{0}^{3}e^{i\left(k_{3\omega }L+3\phi_{\omega }\right)}\frac{1-e^{-\left(\alpha -i\Delta k\right)L}}{\alpha +i\Delta k}$$

This is the general expression for THG emitted in the transmission of a material through excitation by a plane wave that will be used to evaluate experimental results.

### Experimental Setup

The light source we use to generate harmonics is a Ti:sapphire laser (up to 7 mJ, 35 fs, 1 kHz), pumping an optical parametric amplifier (OPA) with 5 mJ of its total output (*TOPAS* from *Light Conversion*), allowing us to tune the central wavelength of the beam in the near-infrared (NIR). All measurements presented in this paper were performed using a fundamental wavelength of 2090 nm with 80 fs pulse duration, and the third-harmonic order, centered at 694 nm, was recorded.

The OPA output is split into two arms: one is used to generate and image harmonic light emitted in transmission from a patterned silicon sample, while the other one is used to produce a reference plane wave from a BBO crystal. Both arms are recombined on a CMOS detector by using a 50/50 fused silica beamsplitter. Figure 1 provides a schematic of the complete experimental setup.

**Figure 1:**
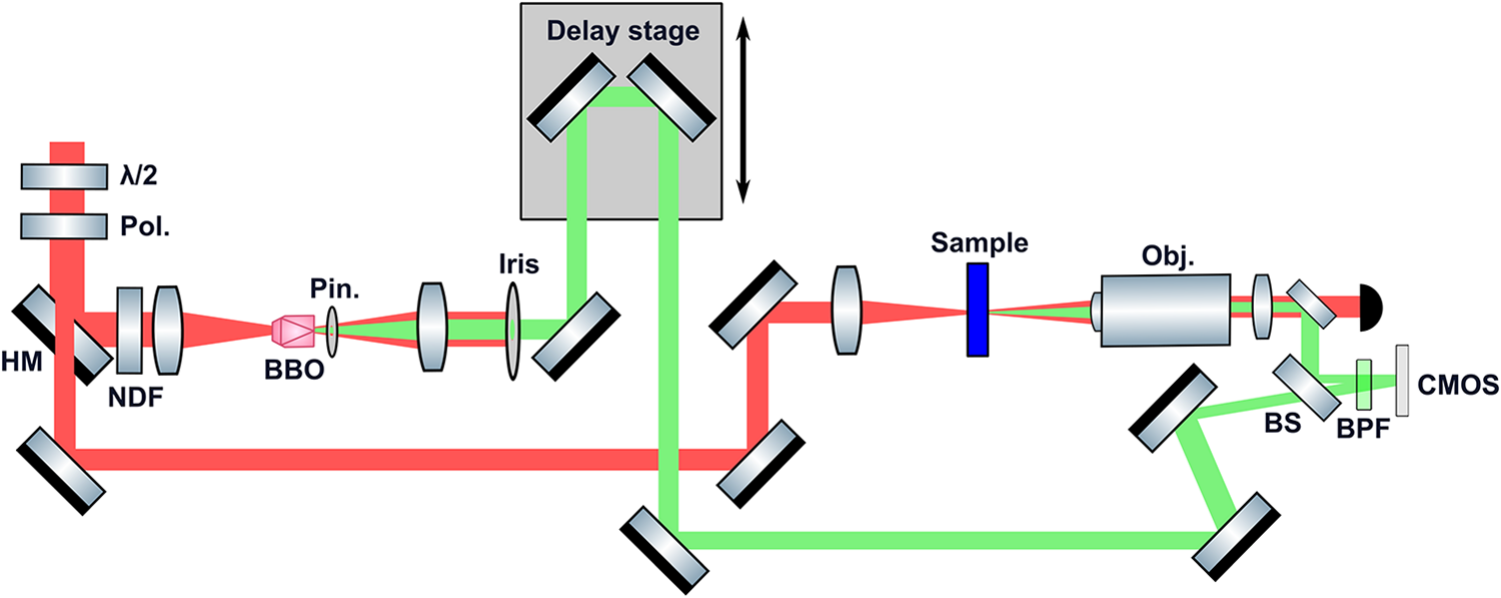
OPA output goes through both a half-waveplate (λ/2) and a thin film polarizer (Pol.) in order to adjust power and polarization. A holey mirror (HM) is used to split the beam in two. The reflected beam, shaped as a donut in the far field, passes through a neutral density filter (NDF) in order to tune its relative intensity with respect to the other arm. It is focused on a BBO in which harmonic generation takes place. A 200 μm diameter pinhole (Pin.) is placed just after the BBO in order to clean up the beam profile in the far field. The donut-shaped NIR fundamental is filtered out using a partially closed iris while the third harmonic is let pass. The beam passes through a delay stage to adjust the time overlap of the two arms. Finally, the beam is sent to the detector by being transmitted through a beamsplitter (BS). The second beam path transmitted from the holey mirror is focused on the sample, and the scattered light is collected by an objective coupled to a tube lens. It is finally reflected from the beamsplitter and reaches the CMOS detector. A bandpass filter (BPF) is placed between the beamsplitter and the detector in order to filter out the fundamental.

The laser is focused onto the sample to a spot size of around 200 μm using a 20 cm focal length *CaF*_2_ lens. This corresponds to a fluence on the order of 20 mJ/cm^2^ and a peak intensity of 3.5 × 10^11^W/cm^2^. This value was chosen in order to maximize third-harmonic emission while staying well below the damage threshold of the material, which we estimated to be close to 100 mJ/cm^2^ or 1.3 × 10^12^W/cm^2^ for the given experimental parameters, where damage here refers to any measurable change in the optical signals after an extended period of exposure. No active stabilization system was needed, as the interferometer displayed a sufficient fringe stability for the time of a hologram recording. Collection of the light was done using a Nikon plan fluorite objective with 0.5 NA, 20× magnification. Fundamental light was removed by inserting an interferometric bandpass filter upon recombination of the two arms.

### Sample Design

The sample we used is a crystalline silicon resolution test target provided by ASML. Etching was performed with e-beam lithography, and all patterns were etched at the same depth of 75 nm (see profilometer measurement presented in Figure 2). The total width of the sample is 700 μm, making it almost completely transparent to the 2090 nm fundamental and partially transparent to the third harmonic, centered at a wavelength of 694 nm. As detailed in the papers from Yamada et al.^22^ and Journigan et al.,^23^ harmonic generation over such a long propagation distance inside silicon is not optimal for conversion efficiency, with effects of nonlinear propagation of the fundamental decreasing the coherent emission of harmonics. Nevertheless, we could obtain high enough yields to allow for hologram recording in relatively short acquisition times (typically one second).

**Figure 2:**
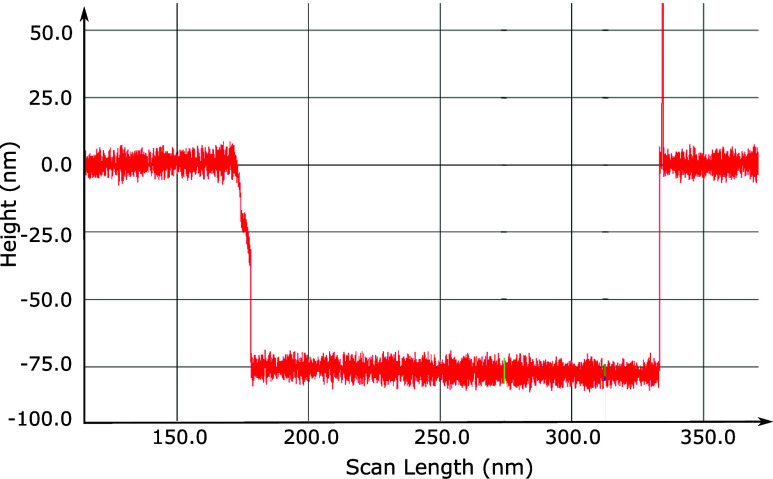
Profilometer measurement of a feature etched in the silicon sample from which the 75 nm height was extracted.

### Hologram Amplitude and Phase Reconstruction Procedure

The numerical procedure used to extract phase and amplitude for harmonic DHM reconstructions from the hologram is essentially the same as that for linear DHM (see Figure 3). The complex-valued electric field in the plane of the detector can be expressed as follows:

8 E(x,y)=S(x,y)+R(x,y)

with *S*(*x*, *y*) being the THG field emitted from the sample and *R*(*x*, *y*) the reference plane wave emitted from the BBO, which can be expressed as *R*(*x*, *y*) = |*R*|e^*i*(*k*_*x*_ *x* + *k*_*y*_ *y*)^. The hologram recorded by the CMOS detector (Figure 3a) is proportional to the intensity of this field

9 $$\begin{array}{l} \left|E\left(x,y\right){|}^{2}=\right|S\left(x,y\right)+R\left(x,y\right){|}^{2}\\ =\left|S\left(x,y\right){|}^{2}+\right|R\left(x,y\right){|}^{2}+S^{*}\left(x,y\right)R\left(x,y\right)+S\left(x,y\right)R^{*}\left(x,y\right) \end{array}$$

**Figure 3:**
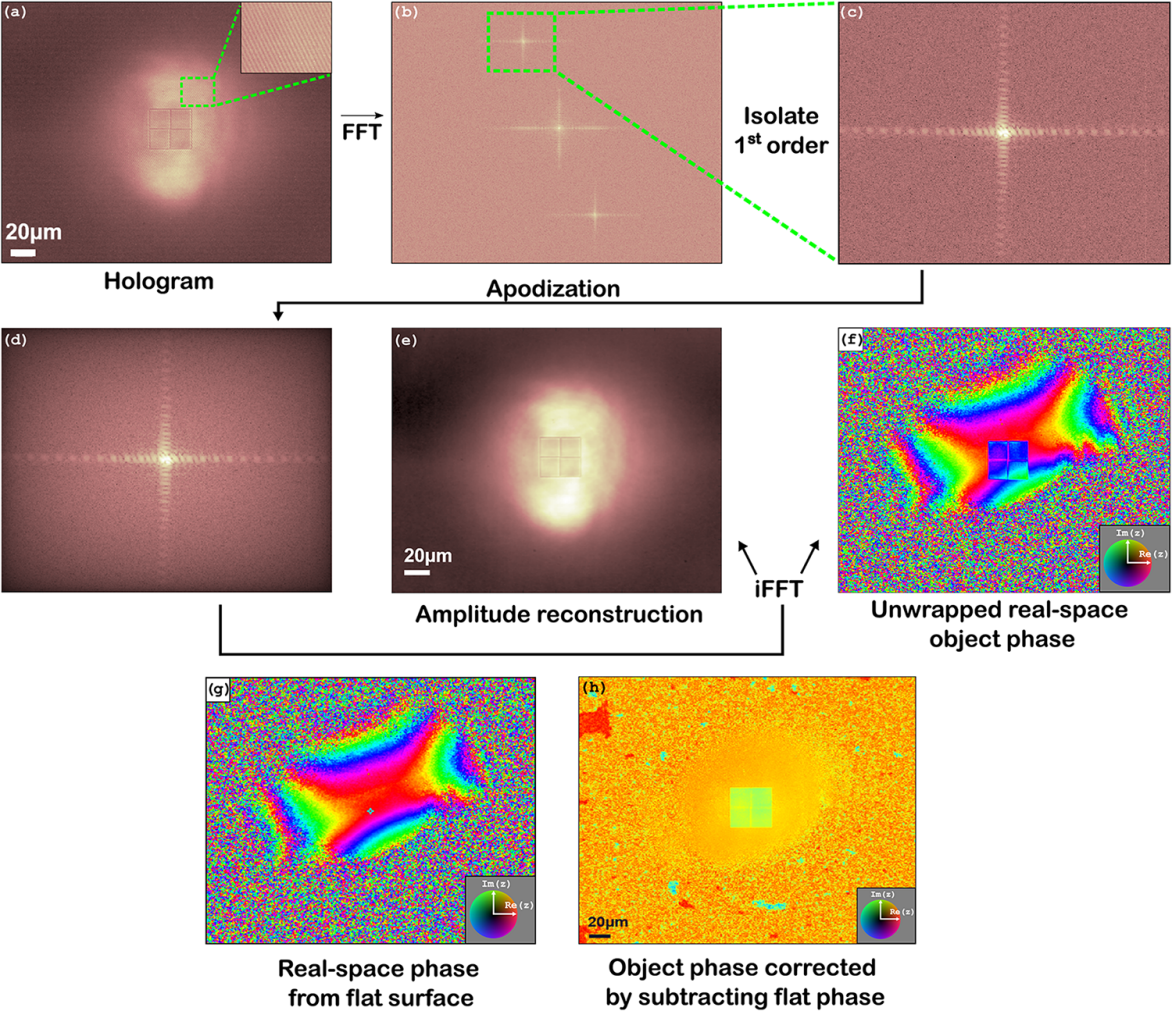
(a) Hologram displaying interference fringes between the sample and reference wave, as shown in the inset. (b) Fourier transform of (a) displaying characteristic DHM convolution terms marked by the green box. (c) Cropped DHM convolution term highlighted from (b). (d) Blackman window apodization of (c). (e) Absolute value of the inverse Fourier transform of (d). (f) Complex phase of the inverse Fourier transform of (d). (g) Phase reconstruction of a flat area on the sample. (h) Subtraction of (g) from (f).

Taking the Fourier transform of this expression yields (Figure 3b)^6,24^

10 $$F^{-1}\left[|E\left(x,y\right){|}^{2}\right]=Ŝ\left(f_{x},f_{y}\right){Ŝ}^{*}\left(f_{x},f_{y}\right)+\left|R\right|\delta \left(f_{x},f_{y}\right)+\left|R\right|Ŝ\left(f_{x}+k_{x},f_{y}+k_{y}\right)+\left|R\right|{Ŝ}^{*}\left(f_{x}-k_{x},f_{y}-k_{y}\right)$$

where hats denote a Fourier transform and *f*_*x*_ and *f*_*y*_ are the frequency space coordinates. The first two terms of this expression correspond to the autocorrelation of both the reference and sample wave Fourier transforms, centered on the origin of the frequency axis. The third term is the Fourier transform of the sample wave convoluted by the Fourier transform of the plane wave reference (which translates to a delta function in frequency space). This convolution allows the shift of the diffraction pattern from the origin and isolation of it from the other terms. The fourth term is the complex-conjugate of the third term, centrosymmetrically flipped with respect to the origin.

The distance of these last two terms from the k-space origin depends on the hologram fringes’ spatial frequency, thus on both the wavelength and the relative incidence angle between sample and reference waves on the camera. If this frequency is too high, the convolution is shifted out of the limits of the accessible frequency window. If the fringe frequency is too low, there is a risk that the pattern overlaps with the terms sitting close to the origin, thus preventing an accurate reconstruction.

By isolating one of these twin patterns (Figure 3c) and taking its inverse Fourier transform, both the amplitude and the phase of the THG field can be obtained

11 $$F^{-1}\left[\left|R\right|Ŝ\left(f_{x},f_{y}\right)\right]=\left|R∥S\left(x,y\right)\right|e^{i\phi \left(x,y\right)}$$

Aberration correction is performed by applying a Blackman apodization filter on top of the isolated pattern in order to remove artifacts arising from cropping abruptly in Fourier space (Figure 3d),^25,26^ after which the inverse Fourier transform in eq 11 is applied to reconstruct amplitude (Figure 3e) and phase (Figure 3f) of the object. As clearly visible in the wrapped phase reconstruction in Figure 3f, the wavefront of the beam illuminating the sample is not flat and gets in the way of obtaining an accurate phase profile. Generally speaking, any deviation from the ideal case of using perfect plane waves for both the sample and reference waves alters the content of the phase and amplitude recovered in eq 11. Such aberrations can be corrected for by measuring a hologram on a flat part of the sample near the pattern of interest (Figure 3g) and using its phase and amplitude reconstructions as references. Finally, a 2D unwrapping algorithm^27^ is used to obtain a continuous phase profile (Figure 3h).

## Results and Discussion

The spatial resolution of the phase reconstructions was determined using the one-image Fourier ring correlation (FRC) method.^28^ Figure 4a–c shows phase reconstruction of multiple patterns etched on the silicon sample, and Figure 4d, their corresponding FRC calculation. The patterns are an array of holes (Figure 4a), a random distribution of particles (Figure 4b), and a resolution target (Figure 4c). According to the 1/7 cutoff frequency criteria,^29^ a resolution of 1.92 μm was extracted (Figure 4d), which is a lesser resolution than what should be achievable at an emission wavelength of 694 nm with a NA of 0.5. We attribute this result to the cropping of the convolution term in Fourier space, which limits the achievable resolution in the final reconstruction.

**Figure 4:**
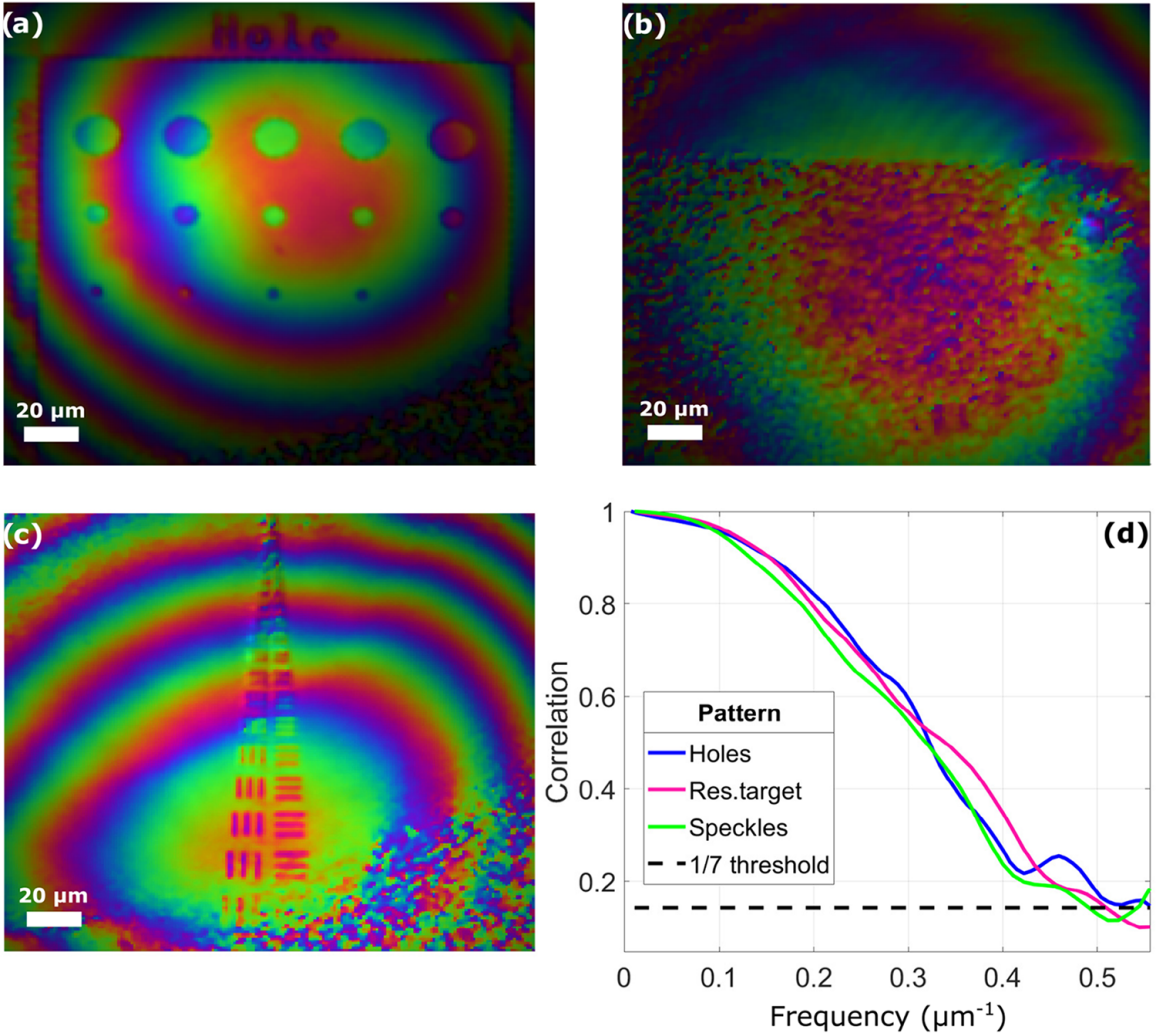
(a) Phase reconstruction of a pattern made of holes. (b) Phase reconstruction of a random particle distribution. (c) Phase reconstruction of a resolution target. (d) FRC calculations using phase reconstructions (a), (b), and (c).

We now delve deeper into analyzing the reconstructed phases. Figure 5 displays the results obtained in the measurement of a pattern composed of multiple holes (Figure 5a). A phase reconstruction (Figure 5b) is obtained from the hologram, the flat phase profile is subtracted from it, and the unwrapping algorithm is applied (Figure 5c). The goal of this measurement is to verify how the phase difference obtained from the hologram relates to the height difference of 75 nm and how it might differ from a linear DHM measurement. The phase line profile in Figure 5d shows that the value is almost constant for both tops and bottoms of the holes. The average phase difference between the etched and pristine parts is 1.82 radians.

**Figure 5:**
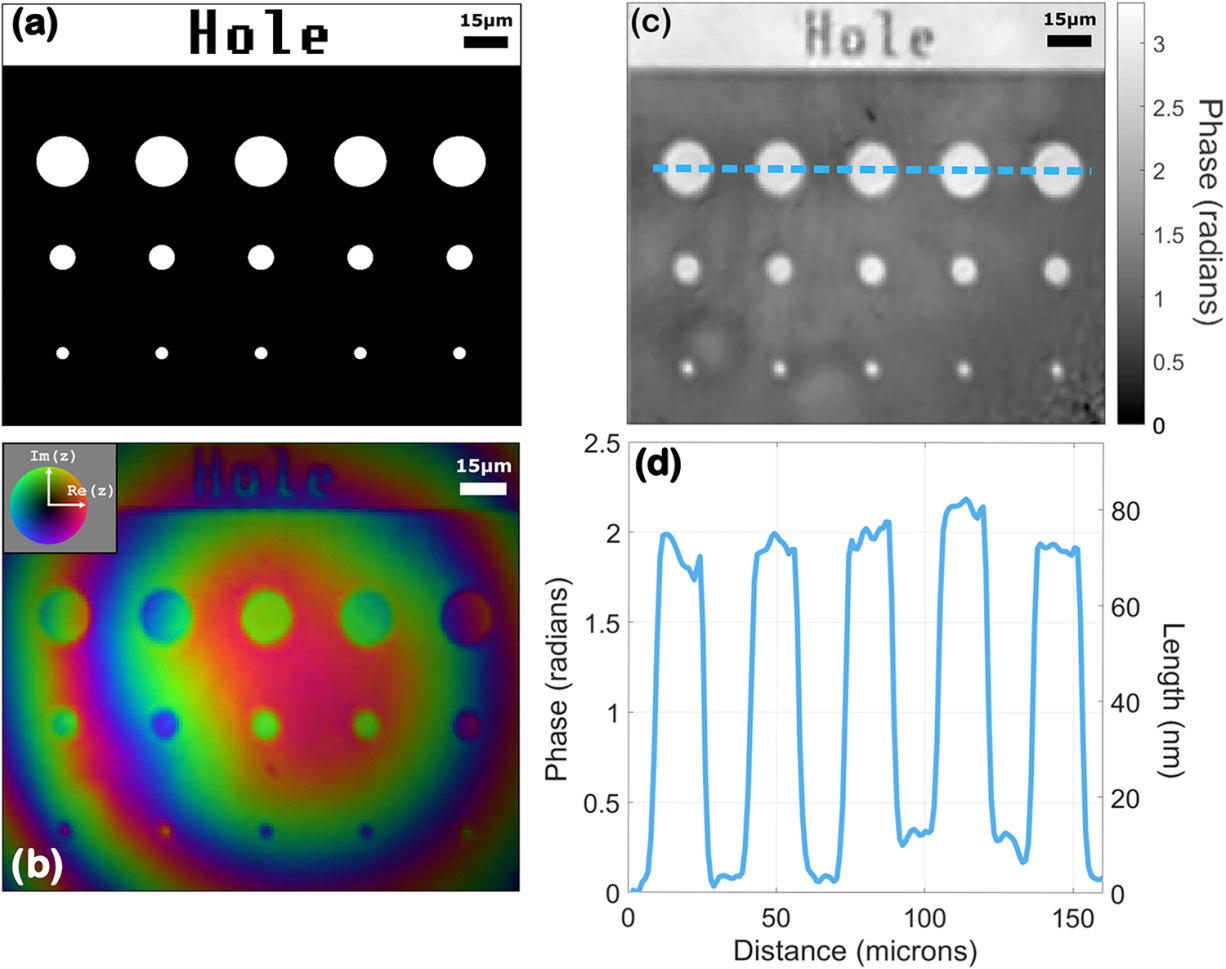
(a) Printing pattern for an array of 75 nm deep holes. The first line holes have a diameter of 16 μm, the second a diameter of 8 μm, and the third a diameter of 4 μm. (b) Reconstruction before subtraction of the reference flat phase. Color encodes phase, and brightness encodes intensity. (c) Unwrapped phase reconstruction with subtraction of the flat phase. (d) Phase profile of the dotted blue line in panel (c).

To easily understand this result, we can separate the THG field into two contributions: a bulk part, which is common to both etched and unetched areas of the sample, and an extra contribution due to the additional layer of the unetched areas

12 $$E_{3\omega }^{\mathrm{tot}}=E_{3\omega }^{\mathrm{bulk}}+E_{3\omega }^{\mathrm{extra}}$$

Although these fields can be derived exactly using eq 5, the absorption length of silicon at 690 nm is around 4.25 μm, which means that the bulk contribution is much larger than the TH contribution from the extra layer of 75 nm. If we assume the phase and amplitude profile of THG is the same for etched and unetched areas at the interface with the nanostructure, we can therefore simplify the problem to the case of a plane wave propagating in two different media. In other words, the pattern essentially acts as a phase plate, altering the propagation of the third harmonic already generated in the bulk. The formula for determining the phase offset between two waves propagating in different media is given by^16,30^

13 $$\Delta \phi =\frac{2\pi }{\lambda }\Delta n\left(\lambda \right)L$$

where λ is the wavelength of the third harmonic, Δ*n*(λ) is the difference in refractive index between air and silicon, and *L* is the depth of the holes.

By averaging the phase difference obtained from multiple holograms, a depth *L* of 71.9 nm is extracted using eq 13, and 71.5 nm is extracted using the general formula given in eq 7, which is in very good agreement with the profilometer measurement. This result confirms that eq 13 holds for harmonic emission from diffracting structures under the condition that the emission is governed by a contribution from the bulk, and not from the nanostructures themselves.

We now turn to the cases where the simple expression used above does not work anymore and focus on subwavelength features. The first set of subwavelength patterns we measured is composed of multiple gratings all having an equal pitch of 600 nm (Figure 6a), with a fill ratio ranging from 10 to 50%, i.e., with critical dimensions ranging from 60 to 300 nm. The reason we chose a slightly subwavelength pitch compared to our emission wavelength (694 nm) was to test approaches such as the effective medium theory, which usually work well for deep subwavelength features and have been successfully applied to, e.g., describing deep subwavelength metasurfaces.^31^ As can be seen in Figure 6b, the lines of the subwavelength gratings are not resolvable in the reconstruction, and a homogeneous phase distribution is measured over the entire extent of the nanostructure after flat phase subtraction (Figure 6c), but a clear edge from the grating is visible. The phase between flat and nanostructured parts of the sample is fitted to a sigmoid function (Figure 6d) so that a phase difference can be extracted for all fill ratios (Figure 6e). We can observe that the phase difference seems to scale exponentially with the inverse of the fill ratio. We find that this result can still be explained—even though the features are relatively close in size to the wavelength—using an effective medium approach: by attributing a fill ratio-dependent effective refractive index to the nanostructure, we can model how the recovered phase behaves. If light is polarized perpendicularly to the grating lines, effective medium theory gives an approximate expression for the complex-valued refractive index^31^

14 $$n_{eff}^{TM}=\epsilon_{a}{\left[\frac{\epsilon_{a}+\frac{1+2FR}{3}\left(\epsilon_{b}-\epsilon_{a}\right)}{\epsilon_{a}+\frac{1-FR}{3}\left(\epsilon_{b}-\epsilon_{a}\right)}\right]}^{1/2}$$

with ϵ_a_ and ϵ_b_ being the refractive indices of both air and silicon at the third-harmonic wavelength (1 for air and 3.80 for silicon), and FR being the fill ratio. The effective refractive index is included in both the general formula for transmission THG (eq 7) and the bulk predominance approximation (eq 13), in order to calculate the expected phase difference as a function of the fill ratio. Figure 6e shows that both models are in very good agreement with the experimental data, confirming that an effective medium approach can be used to extract information on the critical dimension of subwavelength gratings using harmonic holography.

**Figure 6:**
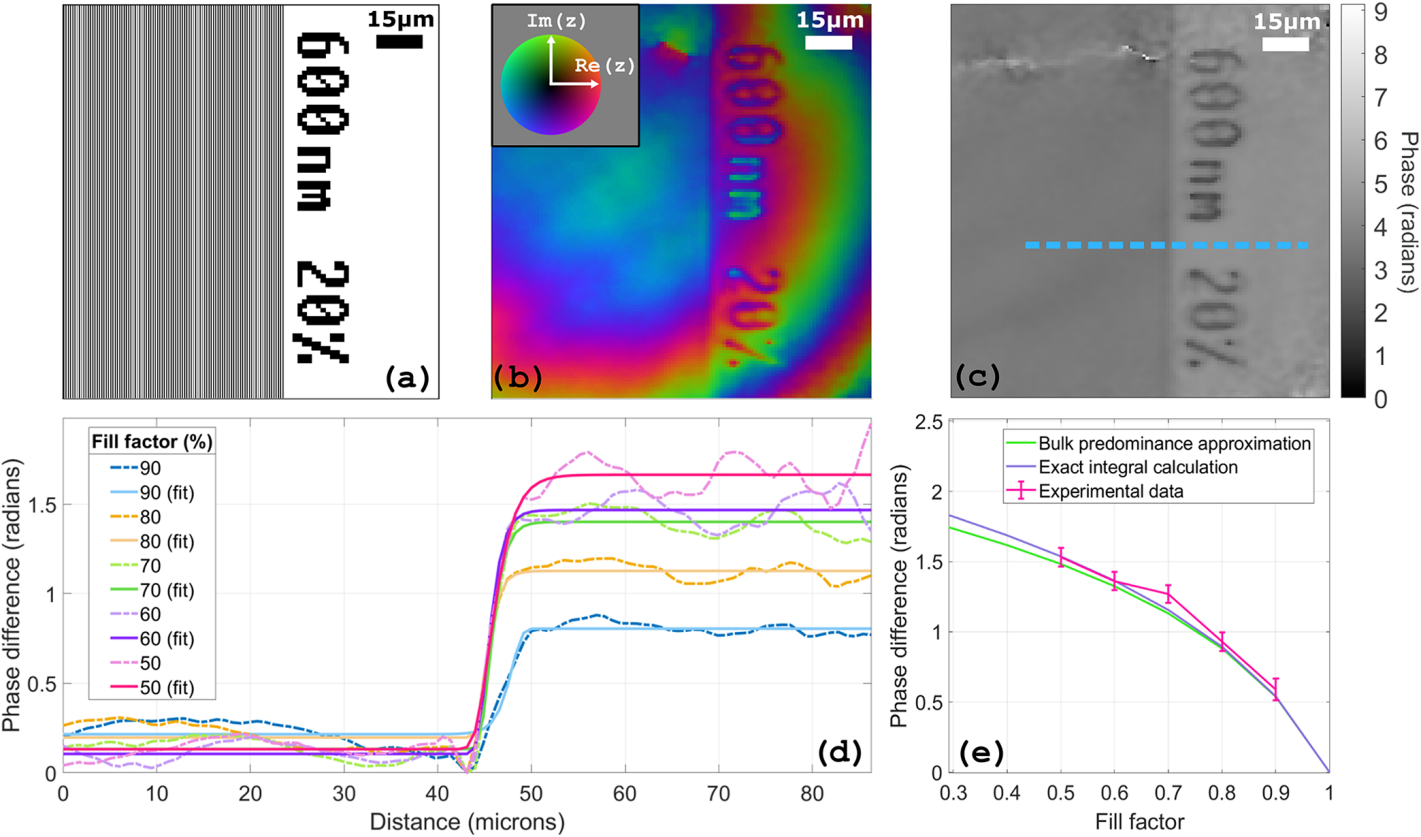
(a) Printing pattern for a subwavelength grating of 600 nm. (b) Reconstruction before subtraction of the reference flat phase. Color encodes phase, and brightness encodes intensity. (c) Unwrapped phase reconstruction with subtraction of the flat phase. (d) Phase profile of the dotted blue line in panel (c) for different fill ratios. Curves are fitted to a sigmoid function to extract a phase difference between flat and subwavelength areas. (e) Phase differences as a function of fill factor.

The second set of nanostructures we focused on is a group of large pitch gratings (20 μm) (Figure 7a), subsegmented into smaller pitch lines (Figure 7b). This design makes it easy to compare the phase from flat parts with the one of subwavelength gratings on a large area of the sample. Inner lines have a constant fill ratio of 50% for all gratings of the set and pitches ranging from 400 to 2000 nm. The depth of the etched lines is 75 nm. As can be seen in the wrapped (Figure 7c) and unwrapped (Figure 7d) phase reconstructions, the 10 μm pitch lines are easily distinguishable, whereas the inner lines are not. This is the case for all pitches of the set, which makes all of them “subwavelength” in the sense of being out of reach of the resolution imposed by our diffraction-limited optical system. Phase profiles are extracted, as shown in Figure 7e, and the average phase difference is calculated. The plot in Figure 7f shows an apparent inversely proportional relationship between the pitch and the phase difference measured between the structured and flat areas. The effective medium approach cannot account for such results, as only the fill ratio should matter, i.e., the effective medium theory would not predict a change in phase as a function of pitch for a constant fill ratio.

**Figure 7:**
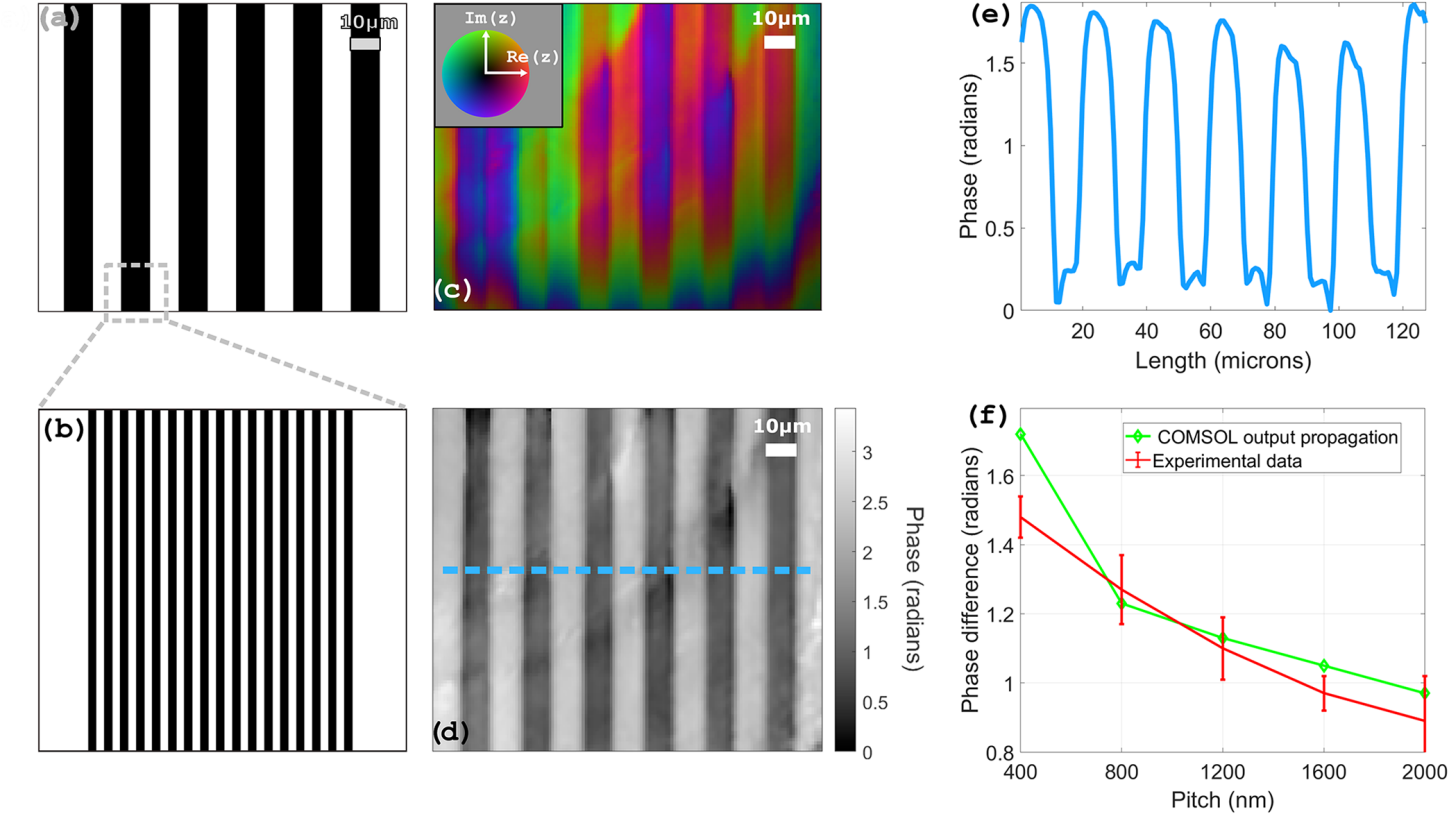
(a) Printing pattern of a subsegmented large pitch grating of 10 μm. (b) Close-up on the subwavelength inner lines, here for a pitch of 600 nm. (c) Reconstruction before subtraction of the reference flat phase. Color encodes phase, and brightness encodes intensity. (d) Unwrapped phase reconstruction with subtraction of the flat phase. (e) Phase profile of the dotted blue line in panel (d). (f) Average phase difference between subwavelength gratings and flat areas for different pitch values.

In order to understand this result, calculations were performed using the electromagnetic waves package of the COMSOL software, which relies on the finite-difference method to solve Maxwell equations.^32^ Our goal was to obtain the third-harmonic wavefront emitted from the sample from the fundamental field distribution, propagate it to the far field, and numerically simulate the measurement in order to compare it with our experimental results, following the approaches in refs 16,33 where the same framework was applied to describe harmonic generation from solids.

Due to the symmetry invariance of the problem, the near-field distribution of a single unit cell, consisting of a 2D cross section in the plane orthogonal to the lines, is enough to establish the distribution of the field in the whole grating (Figure 8a). The input field was simplified to a plane wave at the NIR pulse central wavelength, propagating from the bottom to the top of the unit cell. To obtain the emitted third harmonic, we use the following equation, which accounts for the inhomogeneous distribution of the fundamental:

15 $$E_{3\omega }\left(x,z=L\right)\propto \int_{0}^{L}|E_{0}\left(x,z\right){|}^{3}e^{ik_{3\omega }\left(L-z\right)}e^{-\alpha \left(L-z\right)}dz$$

where *L* is the coordinate of the silicon-air interface along the wave propagation axis and 0 is the coordinate from which the THG contribution is summed up in the silicon bulk. As can be seen in Figure 8b, both phase and amplitude profiles of the third-harmonic wavefront are strongly distorted by the inhomogeneous distribution of the fundamental field. This approach can seem in contradiction with the approximation made earlier in which the TH field generated in the nanostructure was neglected. However, near-field computations confirmed that coupling of the ω field to the subwavelength structures creates some significant change to the intensity distribution on a scale much larger than the etching height (see Figure 8a), therefore explaining why this approximation no longer holds true.

**Figure 8:**
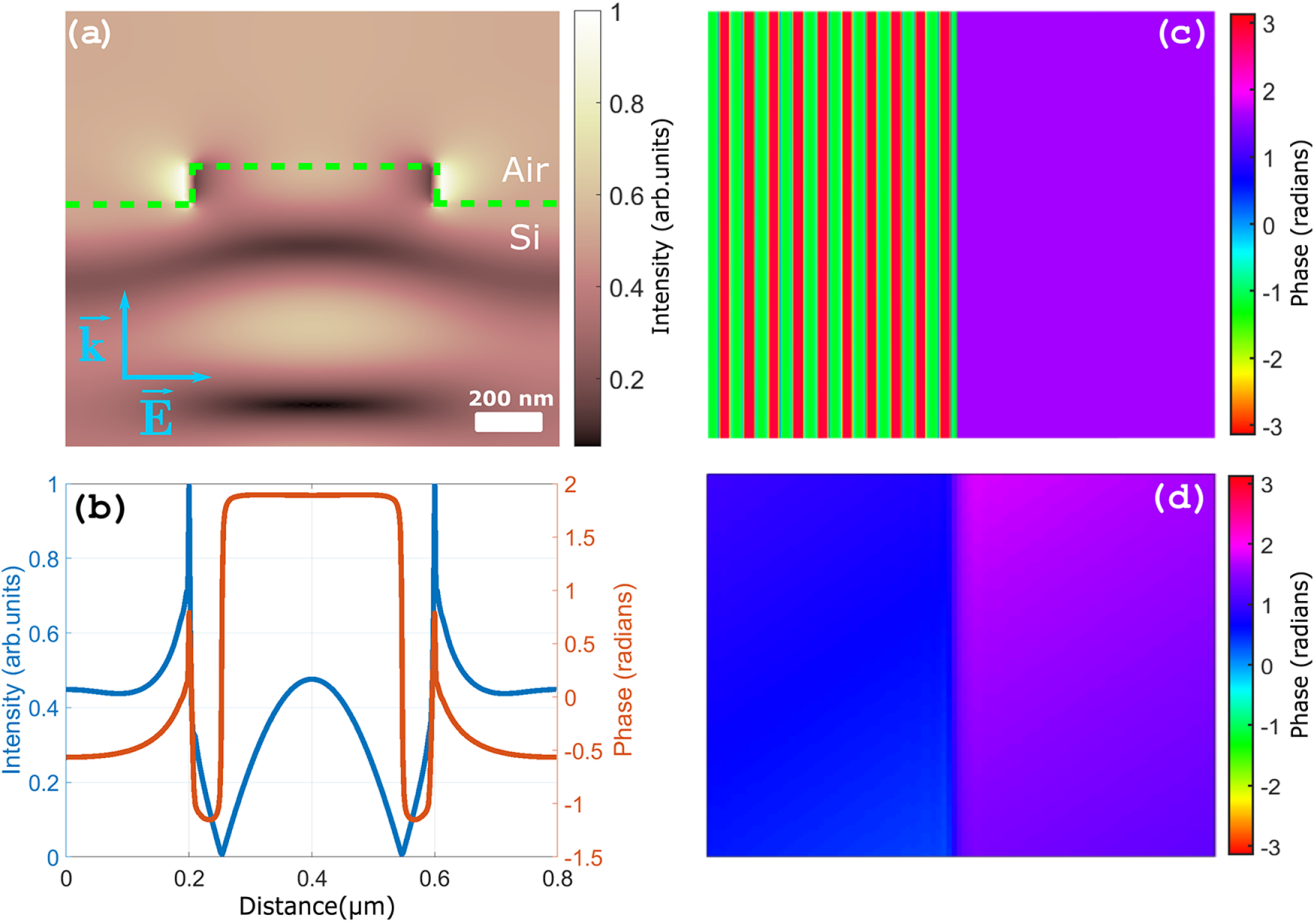
(a) Near-field intensity distribution in a single-line unit cell of 800 μm pitch, obtained using COMSOL software. The input field is a plane wave at the fundamental frequency propagating from the bottom to the top of the plot. (b) Near-field third-harmonic phase and amplitude profile calculated by applying eq 15 to the field distribution in panel (a). (c) Simulated 2D phase distribution of the grating using the unit-cell calculations, juxtaposed to a flat area with no structures. (d) DHM phase reconstruction of panel (c). Details are described in the text.

To simulate the DHM measurement, several of these 1D phase and amplitude profiles are merged to create a grating and then extended in 2D (Figure 8c). A frequency cutoff, corresponding to the NA of the imaging system that was used in the experiment, is applied in Fourier space to replicate the image recorded by the camera, thus making the lines invisible. A plane wave is overlapped on top to form a hologram, and the procedure presented earlier for phase and amplitude reconstruction is applied (Figure 8d). Finally, the phase difference between the flat and nanostructured areas of the sample is computed and compared to the values obtained experimentally, as can be seen in Figure 7f, directly next to the experimental results. The two plots show an excellent agreement, with a slight offset for the smaller pitch of 400 nm, which we assign to the highest SNR for this particular pitch measurement. These results confirm the validity of our approach at modeling the nonlinear scattering from nanostructures and how it reflects in the DHM phase measurement. A practical application of this reconstruction method would be to perform such calculations for multiple values of a periodic structure’s characteristic features to create a library that would be fitted to the measured data. The recovered phase depends on both pitch, CD, and etching depth of the lines. Thus, using this technique to measure these three parameters at once could lead to potential errors. Disentangling each contribution could be achieved by making measurements at different polarizations, since the local-field enhancement of the fundamental shows very different behavior when polarized perpendicular or parallel to the lines.

From the power law dependence in eq 15, we can expect that higher harmonic orders should allow for a large improvement in sensitivity: Any slight change to the fundamental distribution inside the material should translate to larger observable phase shifts, making this technique extremely promising, without being necessarily required to use smaller wavelengths but instead high nonlinearities. These expectations can be mitigated by the fact that higher-order harmonics generated in the nonperturbative domain are expected to deviate from this power law scaling, but we can nevertheless assume some significant resolution enhancement can be achieved.

## Conclusion

Our results show that harmonic DHM allows one to recover phase information in a reliable manner, and accurate height profiles with a very sharp contrast could be extracted from all measurements. The most notable aspect of this technique concerns the reconstruction of periodic subwavelength structures. We could show that measuring phase instead of amplitude allows for precise recovery of depth, but other key morphological parameters as well. Moreover, the technique makes use of harmonic generation power scaling as a tool for improving sensitivity. These results thus provide a promising route and motivation toward applications in the measurement of metrology markers used in the semiconductor industry.

Another important aspect of this paper is that all of the results were obtained by measuring through the transmission of a 700 μm thick silicon sample. Thus, harmonic holography could be used for the inspection of etched patterns on the backside of opaque samples, which would not be possible otherwise. The reconstructions we obtained do not seem to be altered by propagation of the fundamental through the sample, such as self-focusing, which could be expected when dealing with such high intensities. Measurements in a reflection geometry can also be envisioned and are under consideration for upcoming experiments.

Further work will address reconstructions at higher harmonic orders, measuring smaller-sized patterns, and investigating other morphological parameters of gratings such as side-wall angle or surface roughness.

Moreover, we see a particular advantage of our nonlinear imaging approach for studying the emission phase of solid HHG following photoexcitation when used in a photoexcitation-pump/nonlinear-DHM-probe geometry, as our nonlinear DHM measurements provide a self-referenced measurement against the ground-state emission. Recent studies have indicated strong amplitude changes of solid HHG due to photoinduced insulator-to-metal phase transitions,^34,35^ modification to the dielectric function,^33,36^ and photocarrier-induced electron–hole dephasing,^37–40^ but accompanying phase changes remain largely elusive. At the same time, changes of energy levels by photocarrier excitation^38,41^ and driving-intensity controlled dipole phase contributions^42^ have been shown to shift the phase of solid HHG. Understanding amplitude and phase changes following photoexcitation is relevant for unraveling fundamental ultrafast dynamics in solid materials, just like phase-sensitive HHG spectroscopy and advanced molecular dynamics.^43^ Moreover, measuring phases of HHG from excited materials can help further improve super-resolution harmonic deactivation microscopy (HADES)^10,44^ and solid-state HHG source design.^45^
